# Prevelance of depression and anxiety with their effect on quality of life in chronic kidney disease patients

**DOI:** 10.1038/s41598-022-21873-2

**Published:** 2022-10-21

**Authors:** Sameeha Alshelleh, Abdullah Alhouri, Alaa Taifour, Bilal Abu-Hussein, Faris Alwreikat, Mohammad Abdelghani, Muhannad Badran, Yousef Al-Asa’d, Hussein Alhawari, Ashraf O. Oweis

**Affiliations:** 1 Division of Nephrology, Department of Medicine, The University of Jordan, Amman, Jordan; 2 Division of Medical Oncology, Department of Medicine, Imperial College of London, Charing Cross Hospital, London, UK; 3 Department of Medicine, The University of Jordan, Amman, Jordan; 4 Division of Nephrology, Department of Internal Medicine, Jordan University of Science and Technology, Irbid, Jordan

**Keywords:** Chronic kidney disease, Human behaviour

## Abstract

Chronic kidney disease is one of the most common chronic diseases globally. Many studies have shown it is strongly associated with increased social and psychological problems such as depression and anxiety which are considered as common psychiatric disorders that occur in patients with chronic kidney disease. We investigated the prevalence of depression, anxiety and perception of quality of life in a sample of chronic kidney disease patients at the Jordan University Hospital. We aimed to see any association of the mental health in these patients; mainly depression and anxiety with their quality of life and correlation to socio-demographics or laboratory and metabolic profile of this population. 103 chronic kidney disease patients were interviewed using a questionnaire in the Nephrology outpatient clinics of the Jordan University Hospital, the questionnaire included four sections, the first sections handled socio-demographic data. Also, it contains a brief Clinical and laboratory parameter of our patients. The second part consisted of the 9-item Patient Health Questionnaire (PHQ-9) that used to measure the severity of depression. The third part included the 7-item Generalized Anxiety Disorder (GAD-7) to evaluate the severity of anxiety, the fourth part assessed participants quality of life (QOL) using The World Health Organization Quality of Life, Short Form (WHOQOL-BREF) questionnaire. More than half of the participants have depression and anxiety with a percentage of 58.3% and 50.5%, respectively. There was a negative moderate to strong correlation between depression score and quality of life domains scores (p < 0.001).Only marital status had a significant relationship with depression (p < 0.001).Weak positive correlation between Glomerular Filtration Rate and anxiety score (p = 0.04),with significant positive correlation between lipid profile and anxiety score. There was a negative correlation between anxiety score and quality of life domains scores. Females had higher anxiety score than males (p = 0.27). Patients who do not work had a lower physical functioning score compared to others (p value = 0.024).Patients with higher serum Hemoglobin had higher physical and psychological scores. Anxiety, Depression are common among our chronic kidney disease patients, more interventions are needed to improve the mental health of our patients and their quality of life perception. This kind of study allows us to gain a deeper understanding regarding the effects of chronic kidney disease on psychosocial well-being of those patients, and helps health care providers to put depression, anxiety and Quality of life into consideration when treating patients.

## Introduction

Chronic kidney disease (CKD) is one of the most common non communicable diseases and is a growing global health concern^1^. CKD definition and classification guidelines were introduced by the National Kidney Foundation (NKF) and Kidney Disease Outcomes Quality Initiative (KDOQI) in 2002, and were adopted by the international guideline group Kidney Disease Improving Global Outcomes (KDIGO) in 2004 with modifications^2–4^. According to these guidelines, CKD is defined by the presence of kidney damage or decreased kidney function for at least three months regardless of the cause. Glomerular filtration rate (GFR) is generally considered to be the best index of overall kidney function, and declining GFR is the hallmark of progressive kidney disease. CKD is staged according to the cause, GFR, and albuminuria^5^. Staging helps in risk stratification of the patients and also guides management.

Globally, it is estimated that the prevalence of CKD is 11–13%, and the majority being in stage 3^6^. According to national kidney foundation, CKD is the 8th leading cause of death in the USA^7^. Due to the chronic nature of the disease, many studies have shown that it is strongly associated with increased social and psychological problems such as depression, anxiety and decreased Quality of Life (QoL). The disease’s burden, life-long treatment, dietary modification and high cost of treatment are all factors that contribute to the altered psychological status of CKD patients^1^. Depression and anxiety are considered as one of the most common psychiatric disorders that occur in patients living with CKD^1^ and might lead to functional impairment, suicidal ideation, sleep disorders, immune system compromise and worsening of nutritional status^8^, all of which are responsible for the increased morbidity and mortality in this population. Both depression and anxiety in CKD patients are often under diagnosed and untreated^1^. Few studies have been conducted in Jordan regarding this disease entity especially in the absence of a unified database or registry^9^, and no recent studies in Jordan or in our region investigated the psychological well-being in CKD patients.

The aim of this study is to investigate the prevalence of depression and anxiety and perception of QoL in a sample of CKD patients at the Jordan University Hospital (JUH) and investigate if they are correlated to the socio-demographic characteristics and the metabolic profile of the studied population. This kind of study allows us to gain a deeper understanding regarding the effects of CKD on psychosocial well-being of those patients, and helps health care providers to put depression, anxiety and QoL into consideration when treating CKD patients.

### Study design

This is a cross-sectional study that was conducted at the Nephrology outpatient clinics in JUH to evaluate depression, anxiety, and quality of life among chronic kidney disease patients. Data were collected between June/2018 and March/2020. All patients presented to our nephrology clinics were approached for a written consent to participate in the study, inclusion criteria were: CKD patients (Stages I-V) aged 18 years and above. Exclusion criteria were as follows: patients younger than 18 years of age, patients with cognitive impairments, pregnant women and patients with End Stage Renal Disease (ESRD) on dialysis.

### Ethical considerations

This study was approved by the Institutional Review Board (IRB) of the Jordan University Hospital. Study participants voluntarily accepted to participate and they signed an informed consent before starting the interview by field researchers. The anonymity and confidentiality of the participants were ensured by assigning identification numbers to participants, restricted to the research team. All methods were performed in accordance with the relevant guidelines and regulations.

### Data collection and measures

Data was collected from 103 patients who agreed to participate and gave an informed written consent using a structured, validated and published questionnaire used in the literature. Participants in this study were interviewed face to face by field researchers as their health status may prevent them from answering the questionnaire alone. The interviews were carried out at the waiting rooms of the Nephrology outpatient clinics of JUH. Each interview took about 15 min. In our study we used the GFR staging as the following: Stage 1: GFR > 90 mL/min/1.73 m^2^; stage 2: GFR 60–89 mL/min/1.73 m^2^; stage 3: GFR 30–59 mL/min/1.73 m^2^; stage 4: GFR 15–29 mL/min/1.73 m^2^; stage 5: GFR < 15 mL/min/1.73 m^2^ or treatment by dialysis. GFR was calculated from serum creatinine using the Chronic Kidney Disease Epidemiology Collaboration (CKD-EPI) equation^10^.

The questionnaire included four sections: the first sections handled sociodemographic data such as gender, age, marital status occupation and education level. Also, medical records at JUH and laboratory information were reviewed for each patient and included.

The second part consisted of the Patient Health Questionnaire (PHQ-9) that used to measure the severity of depression. For each question the answers range from (0–3) where 0 means never and 3 means always. The total scores categorized as follows: minimal/no depression (0–4), mild depression (5–9), moderate depression (10–14), or severe depression (15–21)^11^.

The third part included the 7-item Generalized Anxiety Disorder (GAD-7) to evaluate the severity of anxiety, with each item having answers ranging from (0–3), where 0 means never and 3 means always. The total scores are categorized as follows: minimal/no anxiety (0–4), mild anxiety (5–9), moderate anxiety (10–14), or severe anxiety (15–21)^12^.

The fourth part assessed participants QOL using the World Health Organization Quality of Life, Short Form (WHOQOL-BREF) questionnaire which includes 26 questions grouped into four domains: physical, psychological, social relationships and environment; in the 26 questions, two are general questions of quality of life and the others represent each of the 24 facets. The WHOQOL-BREF questionnaire was scored after its administration to the study subjects; the raw scores were transformed to 0–100 scales. Each domain presents five possibilities of responses that follow the Likert scale, from 1 to 5. For the interpretation of the results obtained, higher scores reflect a better quality of life.

### Statistical analysis

The data was entered into and analyzed using the Statistical Package for Social Sciences (SPSS), version 25. P < 0.05 was assigned as the alpha value. Data was expressed as mean ± standard deviation (SD) or as counts (%). Data was assessed for normality using Kolmogorov–Smirnov test, histograms and Q–Q plots. Assumptions for using parametric statistics were satisfactory using Levene test for equal variances.

The effects of sociodemographic descriptive on anxiety (GAD‐7), depression (PHQ‐9) and QOL-BREF domains scores were determined by performing independent samples t-test and Mann Whitney-U for each of the following factors: gender, smoking, marital status, education, use of medications and having chronic disease. A Spearman’s rank-order correlation was run to determine the relationship between age, laboratory test results and the scores of QOL-BREF, anxiety and depression. Also, Spearson's correlation was used to study the correlation between QoL scores of each domain of WHOQOL-BREF and the domains themselves, PHQ9 and GAD7 scores.

## Results

A total of 103 CKD patients (30 CKD stage 2 patients, 44 CKD stage 3 patients, and 29 CKD stage 4 and 5 patients) were included in this study, with a mean age of 66.7 years. Most participants were male (62.1%), educated (96.1%), married (85.4%) and nonsmoker (82.5%). Moreover, 79.6% of our patients had less than 5 chronic diseases and were on 5 medications or more (59.2%). Sociodemographic characteristics, clinical and laboratory parameters according to the CKD stage are presented in Table 1.

**Table 1:** Sociodemographic characteristics, clinical and laboratory parameters according to the stage of CKD.

Variable (number)	All (n = 103)	CK2 (n = 30, 29.1%)	CK3 (n = 44, 42.7%)	CK4 & 5 (n = 29, 28.2%)
Age	66.7 ± 13	60.90 ± 12.7	70.4 ± 8.8	66.9 ± 16.4
GFR	47.4 ± 23.5	77.8 ± 7.9	45 ± 8.9	19.7 ± 5.9
Phosphorus	3.8 ± 3.14	3.3 ± 0.6	4 ± 4.7	3.9 ± 0.5
HBa1c	6.3 ± 1.3	6.4 ± 1.4	6.6 ± 1.2	6.3 ± 1.3
Parathyroid hormone	151 ± 106	77.6 ± 45	132.8 ± 66.9	222.8 ± 134.4
Hemoglobin	12.8 ± 1.8	13.7 ± 1.7	13 ± 1.5	11.7 ± 1.5
Albumin	4.1 ± 0.5	4.1 ± 0.6	4.2 ± 0.4	4 ± 0.5
Calcium	9.4 ± 0.6	9.6 ± 0.44	9.4 ± 0.6	9.2 ± 0.7
Uric acid	6.8 ± 1.7	5.7 ± 1.2	6.8 ± 1.3	7.6 ± 2.3
Vitamin D	30.5 ± 18	28.2 ± 12.4	28.2 ± 17.5	36.3 ± 22.1
Triglycerides	172.6 ± 112.3	149.5 ± 61.8	154.7 ± 79.2	230.7 ± 175
High Density Lipoprotein	42.3 ± 14	47.9 ± 11.6	42.1 ± 11.8	36.2 ± 17.4
Low Density Lipoprotein	101.3 ± 39.4	117.51 ± 38.9	100.3 ± 39.1	84 ± 34
Cholesterol	162.8 ± 44.5	176.6 ± 43.8	157.9 ± 43.8	154.8 ± 44.5
Blood pressure systolic	139.2 ± 16.7	137.6 ± 15	139.5 ± 15.3	140.4 ± 20.4
Blood pressure diastolic	78.9 ± 11.9	83.1 ± 9.7	77.6 ± 11.4	76.6 ± 13.9
**Gender**				
Male	64 (62.1%)	14 (46.7%)	32 (72.7%)	18 (62.1%)
Female	39 (37.9%)	16 (53.3%)	12 (27.3%)	11 (37.9%)
**Job**				
Does not work	29 (28.2%)	10 (33.3%)	11 (25%)	8 (27.6%)
Housewife	17 (16.5%)	5 (16.7%)	7 (15.9%)	5 (17.2%)
Retired	31 (30.1%)	5 (16.7%)	18 (40.9%)	8 (27.6%)
Employee	26 (25.3%)	10 (33.3%)	8 (18.2%)	8 (27.6%)
**Marital Status**				
Single	15 (14.6%)	4 (13.4%)	5 (11.4%)	6 (20.7%)
Married	88 (85.4%)	26 (86.7%)	39 (88.6%)	23 (79.3%)
**Educational level**				
Illiterate	4 (3.9%)	0 (0%)	2 (4.5%)	2 (6.9%)
School Education	45 (43.7%)	13 (43.3%)	15 (34.1%)	17 (58.6%)
Diploma	19 (18.4%)	5 (16.7%)	8 (18.2%)	6 (20.7%)
University Degree	35 (33.9%)	12 (39.9%)	19 (43.1%)	4 (13.8%)
**Smoking**				
No	85 (82.5%)	24 (80%)	34 (77.3%)	27 (93.1%)
Yes	18 (17.5%)	6 (20%)	10 (22.7%)	2 (6.9%)
**Number of comorbidities**				
< 5 Diseases	82 (79.6%)	27 (90%)	34 (77.3%)	21 (72.4%)
≥ 5 Diseases	21 (20.4%)	3 (10%)	10 (22.7%)	8 (27.6%)
**Number of medications**				
< 5 Medications	42 (40.8%)	17 (56.7%)	18 (40.9%)	7 (24.1%)
≥ 5 Medications	61 (59.2%)	13 (43.3%)	26 (59.1%)	22 (75.9%)

Regarding depression and anxiety, more than half of the participants were found to have depression and nearly half of them had anxiety with a percentage of 58.3% and 50.5%, respectively. Degrees of depression and anxiety are showed in Tables 2 and 3, respectively.

**Table 2:** Degree of depression according to CKD stage.

Depression degree	All	CK2	CK3	CK4 & 5
No depression	43 (41.7%)	11 (36.7%)	20 (45.5%)	12 (41.4%)
Mild depression	37 (35.9%)	11 (36.7%)	19 (43.2%)	7 (24.1%)
Moderate depression	17 (16.5%)	6 (20%)	3 (6.8%)	8 (27.6%)
Moderately severe depression	4 (3.9%)	1 (3.3%)	2 (4.5%)	1 (3.4%)
Severe depression	2 (1.9)	1 (3.3%)	0 (0%)	1 (3.4%)

**Table 3:** Degree of Anxiety according to CKD stage.

Anxiety degree	All	CK2	CK3	CK4 & 5
No anxiety	51 (49.5%)	9 (30%)	27 (61.4%)	15 (51.7%)
Mild anxiety	26 (25.2%)	9 (30%)	9 (20.5%)	8 (27.6%)
Moderate anxiety	23 (22.3%)	12 (40%)	6 (13.5%)	5 (17.2%)
Severe anxiety	3 (2.9)	0 (0%)	2 (4.5%)	1 (3.4%)

According to Table 4, there was a negative moderate to strong correlation between depression score and quality of life domains scores (p < 0.001). On the other hand, no significant association was detected between depression score and our patients’ laboratory tests (Table 4). With regard to the correlations between socio-demographic variables and depression; only marital status had a significant relationship (p < 0.001). For instance, single patients had a significantly higher depression score (Mean = 9.53 ± SD = 5.7) compared to married patients (Mean = 5.8 ± SD = 4.3) (data not shown).

**Table 4:** Depression and Anxiety scores correlation.

Variable	Depression score		Anxiety score	
	Correlation Coefficient	P-value	Correlation Coefficient	P-value
Age	0.008	0.94	− 0.13	0.18
GFR	− 0.03	0.76	0.20	**0.047**
Phosphorus	0.13	0.20	0.08	0.415
HBa1c	− 0.05	0.64	− 0.05	0.659
Parathyroid hormone	0.14	0.22	0.07	0.516
Hemoglobin	− 0.11	0.28	0.007	0.946
Albumin	− 0.02	0.86	− 0.09	0.382
Calcium	− 0.07	0.46	− 0.08	0.445
Uric acid	− 0.02	0.82	0.08	0.439
Vitamin D	− 0.06	0.57	0.04	0.736
Triglycerides	− 0.02	0.87	− 0.08	0.425
High Density Lipoprotein	0.07	0.51	0.31	**0.003**
Low Density Lipoprotein	0.08	0.46	0.30	**0.005**
Cholesterol	0.13	0.23	0.25	**0.017**
Blood pressure systolic	− 0.08	0.45	0.11	0.259
Blood pressure diastolic	0.03	0.77	0.16	0.112
Depression score	1	–	0.63	**0.000**
Physical Health	− 0.56	**0.000**	− 0.46	**0.000**
Psychological	− 0.44	**0.000**	− 0.34	**0.000**
Social relationship	− 0.38	**0.000**	− 0.23	**0.017**
Environment	− 0.30	**0.000**	− 0.34	**0.001**
Anxiety score	0.64	**0.000**	1	**–**

Significant values are in bold.

There was a weak positive correlation between GFR and anxiety score (rs = 0.20, p = 0.04) and a negative weak association between creatinine and anxiety score (rs = − 0.21, p = 0.03). Additionally, there was a significant positive correlation between lipid profile and anxiety score (Table 4). Similar to the depression score, there was a negative correlation between anxiety score and quality of life domains scores (Table 4). With regard to the association between anxiety score and sociodemographic characteristics, we found that females had a significantly higher anxiety score (mean = 6.74 ± SD = 4.7) than males (mean = 4.7 ± SD = 3.8), p = 0.27 (data not shown).

According to Table 5, the mean total QoL for each domain were as follows: physical functioning (mean = 60.2 ± SD = 15.7), Psychological functioning (mean = 63.9 ± SD = 13.6), Social functioning (mean = 73 ± SD = 16.9) and Environmental domain (mean = 65.9 ± SD = 12.2). Comparison between CKD stages based on the mean total score of QoL for each domain is shown in Table 5.

**Table 5:** QOL domains according to CKD stage.

Quality of life domains	All (n = 103)	CK2 (n = 30, 29.1%)	CK3 (n = 44, 42.7%)	CK4 & 5 (n = 29, 28.2%)
Physical health	60.2 ± 15.7	62.7 ± 15.5	61.4 ± 14.9	55.9 ± 16.6
Psychological	63.9 ± 13.6	64.4 ± 9.6	67.9 ± 12	57.3 ± 17
Social relationship	73 ± 16.9	75.3 ± 20.1	73.1 ± 12.9	70.4 ± 18.7
Environment	65.9 ± 12.2	62.5 ± 9.4	68.7 ± 11.7	65 ± 14.8

There is also a direct correlation between each of the QoL domains. When a certain domain score increases, the other domains also increase (Table 6).

**Table 6:** QOL domains and correlation with laboratory and clinical data.

Variable	Physical health		Psychological		Social relationship		Environment	
	Correlation coefficient	P-value	Correlation coefficient	P-value	Correlation coefficient	P-value	Correlation coefficient	P-value
Age	− 0.11	0.28	0.03	0.75	− 0.09	0.38	0.22	**0.02**
GFR	0.14	0.14	0.20	**0.04**	0.10	0.32	− 0.10	0.31
Phosphorus	− 0.11	0.28	− 0.36	**0.000**	− 0.03	0.65	− 0.28	**0.004**
HBa1c	− 0.21	0.04	− 0.06	0.56	− 0.03	0.76	− 0.004	0.97
Parathyroid hormone	− 0.17	0.13	− 0.16	0.15	− 0.05	0.67	− 0.03	0.77
Hemoglobin	0.25	**0.01**	0.28	**0.004**	0.06	0.56	− 0.07	0.48
Albumin	0.11	0.28	0.21	**0.03**	0.05	0.62	− 0.15	0.14
Calcium	0.17	0.09	0.20	**0.04**	0.12	0.26	− 0.04	0.71
Uric acid	− 0.08	0.45	0.03	0.77	− 0.04	0.74	0.007	0.95
Vitamin D	− 0.07	0.54	0.01	0.93	− 0.12	0.26	− 0.03	0.67
Triglycerides	− 0.04	0.72	− 0.13	0.24	0.09	0.46	0.05	0.63
HDL	0.04	0.68	0.10	0.32	0.06	0.56	− 0.02	0.87
LDL	0.02	0.85	− 0.11	0.29	0.14	0.18	0.02	0.86
Cholesterol	− 0.004	0.97	− 0.15	0.14	0.12	0.25	− 0.03	0.74
Blood pressure systolic	0.04	0.67	0.04	0.66	− 0.06	0.54	0.03	0.76
Blood pressure diastolic	0.09	0.37	− 0.03	0.74	0.08	0.40	− 0.10	0.29
Physical Health	1	**–**	0.43	**0.000**	0.33	**0.001**	0.29	**0.003**
Psychological	0.43	**0.000**	1	**–**	0.29	**0.003**	0.39	**0.000**
Social relationship	0.33	**0.001**	0.29	**0.003**	1	–	0.15	0.12
Environment	0.29	**0.003**	0.39	**0.000**	0.15	0.12	1	**–**

Significant values are in bold.

In addition, there is a weak positive correlation between physical and psychological domains and serum Hb levels as shown in Table 6. Patients who have higher serum Hb levels had higher physical and psychological domain scores.

This study showed that, patients who do not work had a lower physical functioning score (mean = 52.7) compared to the housewives, retired and employee patients (mean = 63, 63.5, 62.8, respectively), p value = 0.024. Additionally, Patients that had university and diploma degree had a higher physical functioning score with a mean score of 65.2, p =  < 0.001 and 66.7, p = 0.001, respectively. However, no relationship was established between illiterate and school educated patients (p = 0.233). Patients that used less than 5 medications had a higher score in the social relationship domain (mean of patients taking less than 5 medications = 77.18, mean of patients taking more than 5 medications = 70.08) p = 0.035. Finally, housewife patients had higher environmental score compared to patients who do not have a job (mean = 72, 61, respectively), p = 0.003 (data not shown).

## Discussion

Psychological wellbeing is an important factor for being healthy. Unfortunately, psychological disorders such as depression and anxiety are usually underestimated or not diagnosed. Our study investigated the prevalence of depression and anxiety and perception of QoL and their correlation with different sociodemographic and metabolic variables.

In our study, we found that the prevalence of depression among CKD patients attending the nephrology clinic at JUH was 58.2%. Our prevalence is similar to several other studies^13,14^, although these studies have used different methods to measure depression. Percentage of patients with moderate to severe depression was 22.3%, a finding that may be comparable to another study by Shafi et al. from Pakistan, which showed a moderate to severe depression percentage of 27%^13^.

According to previously conducted studies, the average lifetime prevalence of depression in the general population has ranged from 14.6% in high-income countries to 11.1% in low-income countries^15^. Also, Razzak et al. has mentioned that the rates of depression in the middle east and north Africa have ranged from 13 to 18%^16^. By comparing these results with the prevalence of depression in CKD patients of 58.2% in our study, a vast difference is noticeable. This can be attributed to the fact that depression is generally affected by numerous factors such as age, gender, chronic diseases and geographical location.

Furthermore, our study has found a significant relationship between depression and quality of life domains including physical activity, psychological, social relationship and environmental domains with p values (0.000, 0.000, 0.000, 0.002) respectively. This is similar to a previous study done on elderly CKD patients which reported significant association between depression and quality of life domain scores which were measured using Kidney Disease Quality of Life-36 (KDQOL-36) instrument^17^. These findings emphasize the close relationship between different aspects of the psychological wellbeing.

Our study has also revealed that depression was significantly higher in single than married patients (p value = 0.004). In our opinion, this can be attributed to the emotional support provided to married CKD patients by their partners.

As for anxiety, it was found to be significantly higher in female CKD patients compared to their male counterparts (p value = 0.027). A previous study has reported a similar finding^18^. This could be explained by the greater sensitivity in women to stressful life events such as diseases. Hormonal differences may play a role as well^19^.

Furthermore, the study has revealed a high prevalence of anxiety (50.4%) among CKD patients attending the nephrology clinics at JUH. Our prevalence of anxiety is less than that found in other studies, in which they reported an anxiety prevalence of 57.1% and 71%^8,20^. This phenomenon can be attributed to the drastic lifestyle changes the chronic kidney disease patients go through. These may include treatment plans, compliance to medications and regular medical tests and procedures as well as dietary modification.

In addition to prevalence, our study has shown an association between anxiety and GFR, in which patients with higher levels of GFR attained higher anxiety score (p value = 0.047). Several researchers argue that the association can be partially attributed to psychological distress that occurs with the declining kidney function and the associated severe changes of the overall health and lifestyle that the patient experience at early stages of disease^21^.

Also, an association has been found between anxiety and different parameters of the lipid profile, several studies have reached similar findings, Goebel et al. performed a study comparing thirty patients suffering from anxiety disorder with thirty controls and concluded that patients with anxiety had elevated serum cholesterol and LDL levels^22^. Dehesh et al. has researched the prevalence and association of anxiety and depression among diabetes patients and found that high anxiety scores correlated with high LDL and high triglyceride levels and came to the conclusion that depression and anxiety may increase the levels of circulating catecholamines and increase the lipoprotein lipase activity leading to a rise in cholesterol and triglyceride levels^23^. Similarly, research has found that patients with pure GAD (general anxiety disorder) have significantly higher cholesterol and triglycerides in comparison to patients with comorbid anxiety and depression concluding that an increase in noradrenergic activity may be responsible for these findings^24^. Multiple researches have also linked anxiety with disturbance in lipid profile levels, this rise in lipid levels lead to a multitude of serious conditions that anxiety was found to complicate and exacerbate including metabolic syndrome and coronary heart disease^24,25^.

Furthermore, a positive correlation was found between anxiety and QoL domains (physical activity, psychological, social relationship and environmental with p values = 0.000, 0.000, 0.017 and 0.001 respectively). As the QoL scores decrease, anxiety scores increase. Few studies have shown similar results. Y-J Lee et al. conducted research studying the relationship between anxiety (measured by the hospital depression and anxiety scale) and quality of life (collected using the WHOQOL-BREF questionnaire) in pre-dialysis CKD patients, which resulted in a negative significant correlation between anxiety and all WHOQOL-BREF domain scores^26^. In another research, Moreira et al. studied the association of anxiety and the quality of life in children and adolescents with pre-dialysis CKD^20^. The results of which showed a negative correlation between anxiety and all of domains of quality of life.

In addition, a positive relationship was found between serum Hb level and both physical and psychological domains, which coincides with other studies^27,28^. In a Study conducted in the US, serum Hemoglobin level had an impact on the QoL in which higher levels result in a better quality of life^29^. This can be attributed to limited oxygen supply associated with low Hemoglobin levels which affect the physical performance^30^.

Our study has also shown a relationship between the psychological domain and both serum albumin and phosphorous levels. An increase in serum Albumin results in higher psychological domain scores, a similar result was found in the literature^31^. Contrarily, Serum phosphorous levels was negatively correlated with the domain, and this was also found in the literature^28^. GFR has been found to have a positive correlation with the psychological domain which is consistent with Chow et al. results^32^.

Regarding the Environmental domain, we found that aging (increase in age) is positively correlated with the domain. This could be attributed to the spiritual practices and religious beliefs of Arab Muslim families which encourage them to provide support to elderly and make them perceive caring for elderly family members as filial piety and a Family obligation^33^.

We also found that depression and anxiety have a negative correlation with QOL. This relationship was reported in a previous study^26^. Similarly, Senanayake et al. has also found the same relationship between depression and QOL^34^.

With regard to Sociodemographic variables, education and job were found to impact QoL, especially the physical domain. Highly educated patients were found to have high physical scores, which is consistent with previous studies^27,28,34^. We believe that highly educated patients show insight and better self-awareness regarding the adherence to treatment plan and the attendance to follow up appointments. In addition, they have higher levels of knowledge and may also live in better economic conditions. Patients who have a Job showed higher physical activity score compared to unemployed patients and Kefale et al., who found similar results, attributes this to better health insurance and financial situation^27^.

## Limitations

The total number of participants (103) were all recruited from Nephrology clinics of Jordan University Hospital. The recruitment of participants from a single medical center has limited our ability to expand the sample size and will probably affect the generalizability of the results. Regarding the CKD stages, the uneven distribution of participants across the four different CKD stage groups has also been a limitation in terms of comparing the prevalence of depression and anxiety among these groups. We hope to conduct a future study including more medical centers in Jordan to cover different geographical areas and increase the number of recruited patients through different stages of CKD.

## Conclusion

Patients with comorbidities, including CKD, are vulnerable to psychological distress. Implementing screening programs or tools within follow up visits can help assess and aid in early diagnosis of psychiatric illnesses, including depression and anxiety. In addition, treating depression and anxiety will improve patients’ Qol given the negative correlation found in the study. We strongly encourage developing psychiatric screening programs for those patients as this will also improve CKD control and health outcomes. Also, we highly recommend increasing awareness towards psychological well-being of CKD patients, especially among nephrologists.

### Ethics approval and consent to participate

This study was approved by the institutional review board at university of Jordan, a written informed consent was obtained from all patients prior to enrollment.

## Data Availability

The datasets used and/or analysed during the current study are available from the corresponding author on reasonable request.

## Abbreviations

CKD: Chronic kidney disease
QoL: Quality of life
GFR: Glomerular filtration rate
JUH: Jordan university hospital
PHQ-9: 9-Item patient health questionnaire
GAD-7: 7-Item generalized anxiety disorder
WHOQOL-BREF: The world health organization quality of life, short form
NKF: National kidney foundation
KDOQI: Kidney disease outcomes quality initiative
KDIGO: Kidney disease improving global outcomes
ESRD: End stage renal disease
IRB: Institutional review board
CKD-EPI: Chronic kidney disease epidemiology collaboration
KDQOL-36: Kidney disease quality of life-36
LDL: Low density lipoprotein
GAD: General anxiety disorder
